# Relationship between glioblastoma location and O^6^-methylguanine-DNA methyltransferase promoter methylation percentage

**DOI:** 10.1093/braincomms/fcae415

**Published:** 2024-12-03

**Authors:** Giulio Sansone, Giuseppe Lombardi, Marta Maccari, Matteo Gaiola, Lorenzo Pini, Giulia Cerretti, Angela Guerriero, Francesco Volpin, Luca Denaro, Maurizio Corbetta, Alessandro Salvalaggio

**Affiliations:** Department of Neuroscience, University of Padova, 35121 Padova, Italy; Department of Oncology, Oncology 1, Veneto Institute of Oncology IOV-IRCCS, 35128 Padova, Italy; Department of Oncology, Oncology 1, Veneto Institute of Oncology IOV-IRCCS, 35128 Padova, Italy; Department of Neuroscience, University of Padova, 35121 Padova, Italy; Department of Neuroscience, University of Padova, 35121 Padova, Italy; Department of Oncology, Oncology 1, Veneto Institute of Oncology IOV-IRCCS, 35128 Padova, Italy; Surgical Pathology and Cytopathology Unit, Department of Medicine-DIMED, University of Padua School of Medicine, 35121 Padova, Italy; Division of Neurosurgery, Azienda Ospedaliera Università di Padova, 35128 Padova, Italy; Academic Neurosurgery, Department of Neurosciences, 35121 University of Padova, Padova, Italy; Department of Neuroscience, University of Padova, 35121 Padova, Italy; Padova Neuroscience Center (PNC), University of Padova, 35121 Padova, Italy; Venetian Institute of Molecular Medicine (VIMM), Fondazione Biomedica, 35129 Padova, Italy; Department of Neuroscience, University of Padova, 35121 Padova, Italy; Padova Neuroscience Center (PNC), University of Padova, 35121 Padova, Italy

**Keywords:** MGMT, MRI, GBM, location, pyrosequencing

## Abstract

A large literature assessed the relationships between the O^6^-methylguanine-DNA methyltransferase (MGMT) promoter methylation status and glioblastoma location with inconsistent results. Studies assessing this association using the percentage of methylation are lacking. This cross-sectional study aimed at investigating relationships between glioblastoma topology and MGMT promoter methylation, both as categorical (presence/absence) and continuous (percentage) status. We included patients with diagnosis of isocitrate dehydrogenase wild-type glioblastoma [World Health Organization (WHO) 2021 classification], available pre-surgical MRI, known MGMT promoter methylation status. Quantitative methylation assessment was obtained through pyrosequencing. Several analyses were performed for categorical and continuous variables (*χ*^2^, *t*-tests, ANOVA and Pearson’s correlations), investigating relationships between MGMT methylation and glioblastoma location in cortex/white matter/deep grey matter nuclei, lobes, left/right hemispheres and functional grey and white matter network templates. Furthermore, we assessed at the voxel-wise level location differences between (i) methylated and unmethylated glioblastomas and (ii) highly and lowly methylated glioblastomas. Lastly, we investigated the linear relationship between glioblastoma-voxel location and the MGMT methylation percentage. Ninety-three patients were included (66 males; mean age: 62.3 ± 11.3 years), and 42 were MGMT methylated. The mean methylation level was 33.9 ± 18.3%. No differences in glioblastoma volume and location were found between MGMT-methylated and MGMT-unmethylated patients. No specific anatomical regions were associated with MGMT methylation at the voxel-wise level. MGMT methylation percentage positively correlated with cortical localization (*R* = 0.36, *P* = 0.021) and negatively with deep grey matter nuclei localization (*R* = −0.35, *P* = 0.025). To summarize, we investigated relationships between MGMT methylation status and glioblastoma location through multiple approaches, including voxel-wise analyses. In conclusion, MGMT promoter methylation percentage positively correlated with cortical glioblastoma location, while no specific anatomical regions were associated with MGMT methylation status.

## Teaser

This is the first study investigating relationships between the glioblastoma location and O^6^-methylguanine-DNA methyltransferase promoter methylation, both as categorical (presence/absence) and continuous (percentage) status. Sansone *et al*. found that the methylation percentage positively correlated with cortical location, while no specific anatomical regions were associated with O^6^-methylguanine-DNA methyltransferase methylation status.

## Graphical Abstract

**Graphical Abstract:**
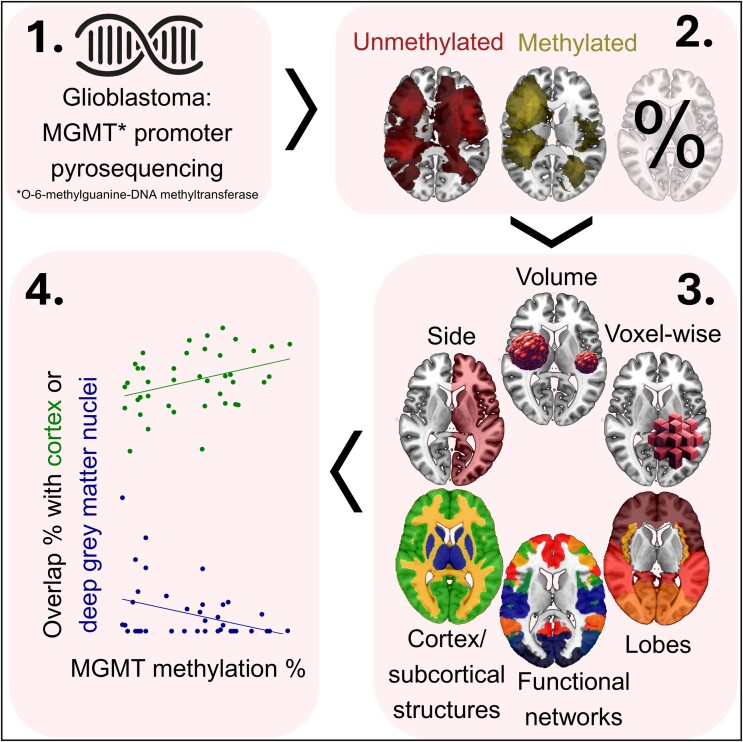

## Introduction

Glioblastoma (GBM) is the most frequent primary central nervous system malignant tumour among adults. Its incidence rate is 3–5 cases per 100 000 people each year,^1,2^ with a median overall survival (OS) of around 15 months.^1,2^ The methylation status of the O^6^-methylguanine-DNA methyltransferase (MGMT) promoter is a crucial prognostic factor in patients with GBM. In particular, when these patients are exposed to chemotherapies with alkylating agent (e.g. temozolomide), the methylation-dependent silencing of MGMT expression allows an increase in the DNA binding of O^6^-alkylguanine, which in turn leads to further genomic damage and cell death, with increased temozolomide efficacy.^3^ Additional critical prognostic factors include pre-surgery performance status, age of diagnosis, the radicality of the surgical resection and radiotherapy or chemotherapy eligibility.^4^ The tumour location does not directly impact patient prognosis unless it influences the extent of the surgical resection.^5^ On the contrary, a recent study from our group showed the relevance of the density of white matter fibre tracts at the site of the GBM lesions as a prognostic factor.^6^

Interestingly, GBMs have been classified not only according to molecular profiling patterns^7^ but also distinct DNA and histone methylation patterns of GBMs.^8,9^ Furthermore, these distinct GBM subtypes have been associated with different anatomical topologies,^9-11^ and there are preliminary evidences that MGMT expression tends to be lower in the glioma CpG island-methylator phenotype and proneural GBM subtypes.^9,12,13^ Hence, several studies have specifically assessed the relationships between MGMT methylation status and GBM location, yet no consistently significant association was reported, and results appear conflicting. In fact, more recent evidence suggests the absence of a clear relationship.^14-24^  *Table 1* summarizes the methods and results of all such studies, including the present one.

**Table 1:** Summary of the studies addressing MGMT promoter methylation and GBM location

Study	Number and type of patients included	Methods used to determine tumour location	Statistics used to investigate relationships between tumour location and MGMT methylation status	Results
Ellingson *et al*., 2012^14^	358 *de novo* GBMs (no IDH mutational status information available)	Voxel-based	Voxel-wise analysis (analysis of differential involvement/ADIFFI statistical mapping)	MGMT-unmethylated GBMs are prevalent in the right hemisphere, while MGMT-methylated GBMs prevail in the left hemisphere, in particular in the temporal lobe
Ellingson *et al*., 2013^15^	507 *de novo* GBMs, including both IDH mutated and wild-type tumours	Voxel-based	Voxel-wise analysis (analysis of differential involvement/ADIFFI statistical mapping)	MGMT-unmethylated tumours are prevalent in the right temporal lobe, while MGMT-methylated ones prevail in the left temporal lobe
Eoli *et al*., 2007^16^	86 patients, including both *de novo* and secondary GBMs	Visual inspection (frontal, temporal and ‘other’, the latter one including parietal and occipital lobes)	*χ* ^2^ and Fisher’s exact tests	MGMT-methylated tumours were more often located in parietal and occipital lobes, while MGMT-unmethylated tumours were more frequently found in the temporal lobes
Wang *et al*., 2014^17^	153 *de novo* GBMs	Voxel-based	Voxel-wise analysis	Low MGMT-expressing GBMs were more likely to occur in the right temporal-parietal lobe, while tumours with high expression of MGMT protein occurred more often in the left frontal lobe
Han *et al*., 2018^18^	92 IDH wild-type *de novo* GBMs	Visual inspection, defining four types of location categories: tumours in which the contrast-enhancing lesion contacts both the SVZ and the cortex; tumours that contact the SVZ but not the cortex; tumours contacting the cortex but not the SVZ; tumours contacting neither the SVZ nor the cortex)	Fisher’s exact tests	MGMT-methylated GBMs more frequently spare the subventricular zone
Drabycz S. *et al*., 2010^19^	59 GBMs, no IDH mutational status info available	Assessment of overlap between lesion centroid and anatomical regions in brain atlas. For each tumour, the authors indicated the mainly involved region (frontal, parietal, temporal, occipital, basal ganglia or cerebellum) and sector (inferior versus superior; left versus right; anterior versus posterior)	Fisher’s exact test	No associations
Carrillo *et al*., 2012^20^	190 GBMs, including IDH mutated and wild-type tumours	Visual inspection (tumour belonging to a certain location was defined based on them being centred in the frontal, parietal, temporal, occipital, insula, posterior fossa, deep grey matter nuclei)	*χ* ^2^ tests	No associations
Roux *et al*., 2019^21^	192 IDH wild-type GBMs	Voxel-based	Voxel-wise analysis	No associations
Incekara *et al*., 2020^22^	436 IDH wild-type GBMs	Voxel-based	Voxel-wise analysis	No associations
Tejada Neyra *et al*., 2018^23^	221 IDH wild-type GBMs	Voxel-based	Voxel-wise analysis	No associations
Sasaki *et al*., 2019^24^	201 IDH wild-type and mutated GBMs	Voxel-based	Least absolute shrinkage and selection operator (LASSO)	No associations
The present study	93 IDH wild-type GBMs (analyses were also repeated flipping the lesions to the same side to virtually reach 186 patients)	Voxel-based Atlas-based definition of side (right versus left), lobe (frontal, temporal, parietal, occipital, insular), compartment (cortex, white matter, deep grey matter nuclei), functional grey and white matter networks	Voxel-wise analysis *χ* ^2^ tests, repeated measures ANOVA, Student’s *t*-tests, Pearson’s correlations	No associations with specific anatomical locations. MGMT methylation percentage positively correlates with cortical involvement and negatively with deep grey matter involvement

MGMT methylation status is most commonly assessed through pyrosequencing and methylation-specific PCR. The latter is a DNA sequencing method used to quantify the methylation level of MGMT promoter-associated CpG islands. The procedure implies the use of a mixture containing deoxy-nucleotides, ATP-sulfurylase, DNA-polymerase and the luciferase enzyme, which is applied to the extracted single-stranded DNA, thus allowing to detect the individually incorporated nucleotide types, through measurement of the resulting light emission.^25,26^ Pyrosequencing has been proven a highly reliable and reproducible technique in several studies, despite the absence of a standardized threshold to distinguish MGMT-methylated from MGMT-unmethylated GBMs.^25,26^

Concerning the prognostic implications of the quantitative determination of the MGMT promoter methylation value, the average methylation extent of some CpG islands significantly correlated with the median OS of patients affected by GBM in three separate works.^27-29^ Furthermore, a recent pyrosequencing-based study showed significantly better outcomes in patients harbouring tumours with higher MGMT methylation percentages.^30^

Nonetheless, there is currently no published data regarding the association between GBM localization and the MGMT promoter methylation percentage. The present study aimed at investigating the relationships between the MGMT promoter methylation status, both as categorical (presence/absence) and continuous status, and GBM location. Moreover, because of the increasing evidence that GBMs have a non-homogenous anatomical distribution across different functional brain networks,^31,32^ we also investigated whether GBM-network topology was related to MGMT promoter methylation.

## Materials and methods

### Patients

This retrospective cross-sectional study was carried out on 112 patients, who were surgically treated between 1 February 2015 and 30 November 2020. The inclusion criteria were as follows: (i) newly diagnosed IDH wild-type GBM (WHO 2021 classification);^33^ (ii) presence of an MRI scan with pre-contrast, post-contrast T1-weighted, T2-weighted and FLAIR sequences, prior to the neurosurgical operation; and (iii) known MGMT methylation status by pyrosequencing. Patients were excluded in the following cases: recurrent disease; magnetic field strength lower than 1.5 T; absence of axial plane acquisition in either pre-contrast, post-contrast T1-weighted or FLAIR sequences; significant MRI artefacts; and previous invasive brain procedures (e.g. biopsy). The present study was approved by the Province of Padua’s ethical committee (protocol no. 70n/AO/20) and carried out in compliance with the Declaration of Helsinki, as well as with its latest amendments.

### MGMT methylation status assessment

For each tissue sample, DNA was analysed through pyrosequencing to quantify the methylation status of 10 CpG sites (CpGs 75–84). Their methylation density was assessed using the ‘PyroMark Q96’ software, and, after computing the mean of all 10 sites, the methylation percentage was calculated for each sample. When data were missing, the analysis was deemed unsatisfactory and would be performed on a different lesion sample. Only GBMs with a methylation percentage ≥ 8% have been considered as MGMT methylated, as this has been used as a reliable cut-off in previous studies.^25,34^ All the technical phases of the procedure are explained thoroughly in a previous paper.^30^

### Pre-processing of MR images

MRI pre-processing first implied image bias field correction^35^ and skull-stripping.^36^ Afterwards, images underwent co-registeration to the corresponding pre-contrast T1-weighted sequence. Subsequently, slice-by-slice manual segmentation in the native space was performed with the ITK-Snap toolbox version 3.8.0 (www.itksnap.org) by two neurology residents (G.S. and M.G.) and verified by an expert neurologist (A.S.). For each tumour, two regions of interest were obtained from the segmentation of four tissue types: tumour core (encompassing volumes of contrast-enhancing tumour, non-contrast-enhancing tumour, necrosis) and oedema. The ensemble of ‘core’ and ‘oedema’ was referred to as ‘global’ volume. The ‘virtual brain grafting’ technique was used to normalize lesion masks.^37^ Details on imaging segmentation and normalization are reported in Salvalaggio *et al*.^38^

### Overlap between brain lobes, cortical, subcortical structures, functional networks and GBM

For each patient, we computed the frequency of GBM core overlap with brain lobes (at least 250 voxels), using the USCLobes atlas (https://brainsuite.org/usclobesatlas/), as well as both the volume of and the percentage of the GBM core volume that overlapped brain cortex, white matter and deep grey matter nuclei (caudate, putamen, globus pallidus, amygdala and nucleus accumbens) using the Harvard-Oxford atlas (https://fsl.fmrib.ox.ac.uk/fsl/fslwiki/Atlases), investigating differences related to the MGMT methylation status. For each patient, we also computed overlap percentages between GBM core lesions and functional brain network atlases, employing resting-state fMRI-derived MRI atlases of both grey and white matter functional brain networks (GMNs and WMNs, respectively). For GMNs, we employed Yeo *et al*.’s parcellation,^39^ which includes 17 subnetworks. To these latter ones, we included the Harvard-Oxford subcortical atlas’ deep grey matter nuclei (basal ganglia and thalami) and hippocampi, as additional parcels. Regarding WMNs, Peer *et al*.’s 12-network parcellation^40^ was employed. In detail, for each core mask, we calculated the ratio between the mask voxels included in a given network and the voxels within all networks of the same tissue (either GMNs or WMNs). For further details regarding the networks and the procedures, see also Sansone *et al*.^32^ Overlap measures were calculated using in-house python scripts.

### Statistical analyses

Beyond descriptive statistics (median, frequencies, mean, standard deviation), the analyses included two-tailed *t*-test and *χ*^2^ test assessing differences in core or oedema volume, in network overlap percentages and in the involvement frequency of cerebral lobes or hemispheres, between MGMT-methylated and MGMT-unmethylated or between highly and lowly methylated tumours. For the latter analyses, we used the median as cut-off. For volume differences, we also subdivided patients into four categories, according to the combination of their MGMT methylation status and side of the involved hemisphere. Additionally, we performed two repeated measures ANOVA models, for GMNs and for WMNs separately, including, for each subject, 19 overlap measures for the latter and 12 measures for the first model. Moreover, Pearson’s correlation tests were also performed between MGMT methylation percentage and core/oedema volumes or GBM core overlap percentages with cortex/white matter/deep grey matter nuclei. The latter ones were also corrected for GBM core volume (partial correlations).

The level of significance was set to 0.05. When multiple comparisons were involved, results were corrected with the Bonferroni correction.

### Voxel-wise analysis

Voxel-wise analyses were carried out with FSL’s general linear model software (https://fsl.fmrib.ox.ac.uk/fsl/fslwiki/GLM) and the ‘randomise’ function (1000 random permutations). Firstly, we performed two-sample *t*-tests assessing voxel-wise differences in GBM core mask location between MGMT-methylated and MGMT-unmethylated tumours. Furthermore, we both investigated linear relationships between MGMT methylation percentage and mask location and assessed location differences between highly and lowly methylated patients, using the median as the cut-off. Furthermore, we repeated voxel-wise analyses after flipping the lesions to the same side, in order to increase the statistical power.

## Results

### GBM patients’ characteristics

Ninety-three patients were included (66 males; mean age: 62.3 ± 11.3 years), 14 were excluded because of MRI artefacts and other 5 were excluded because of the lack of methylation data. Forty-two patients were MGMT-methylated, and the quantitative MGMT promoter methylation quantification with pyrosequencing was carried out in 41 (26 males; mean age: 66.4 ± 8.4 years). The mean methylation level was 33.9 ± 18.3% (median: 37%). GBM core, oedema and global volumes were 43.2 ± 29.3, 54.8 ± 43.4 and 98 ± 52.9 mL, respectively. The frontal lobe was involved in 60 patients (64.5%), the insular lobe in 53 patients (57%), the temporal in 46 (49.5%), the parietal in 66 (71%) and the occipital in 22 (23.7%).

### MGMT promoter methylation and GBM volume, side and lobar involvement

**Figure 1:**
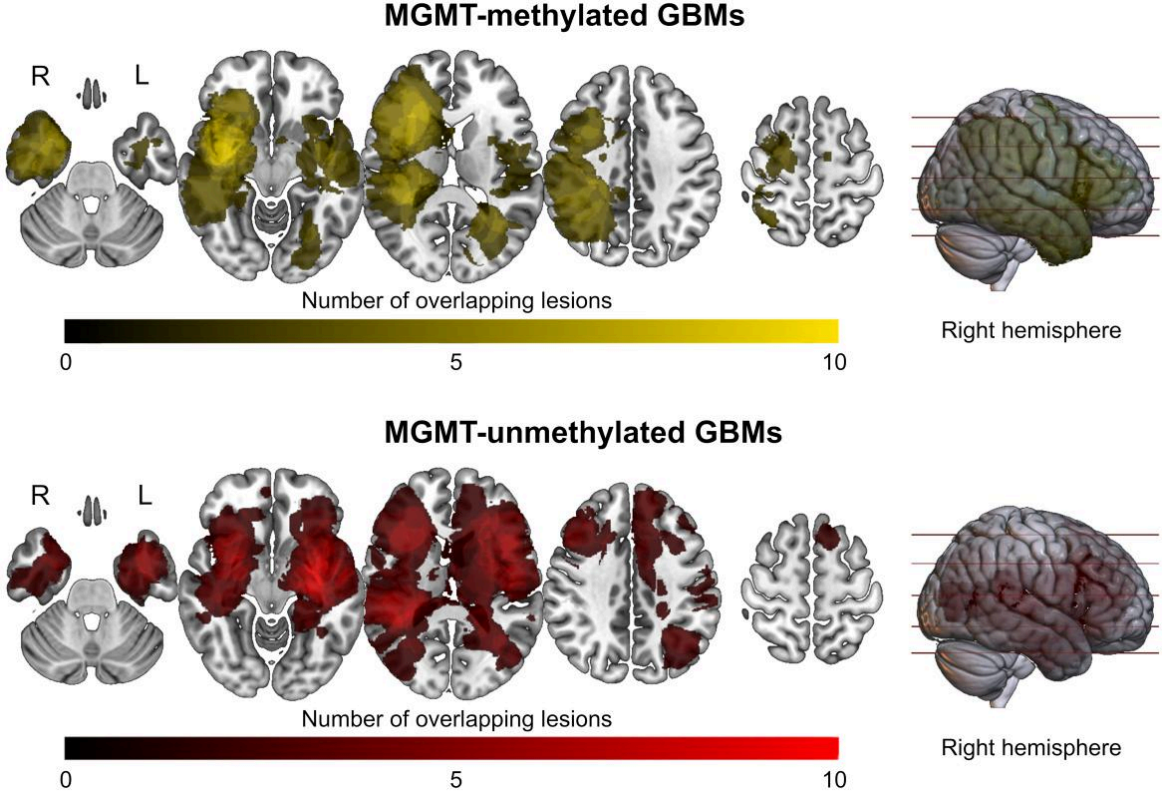
**Distribution frequency map of GBM cores according to the MGMT methylation status.** The map for our sample’s MGMT-methylated GBMs is shown in the top section, while that for MGMT-unmethylated GBMs is shown in the bottom section. The brighter the colour, the higher the number of overlapping GBMs in the corresponding area. Only regions overlapped by at least two GBMs are shown. GBMs, glioblastomas.

There were no statistically significant differences between MGMT-methylated and MGMT-unmethylated GBMs’ core, oedema or global volumes. The only difference in global volume was found between right MGMT-methylated and MGMT-unmethylated GBMs (110.5 mL versus 95.7 mL, *P* = 0.016), even though it lost significance after Bonferroni correction (significance at *P* < 0.008). Among MGMT-methylated GBMs, no significant differences in core, oedema and global sizes between highly and lowly methylated GBMs were found. Moreover, we found no significant correlations between the MGMT promoter methylation percentage and core (*R* = 0.1, *P* = 0.47), oedema (*R* = −0.15, *P* = 0.36) or global volumes (*R* = −0.04, *P* = 0.8). MGMT-methylated GBMs were more frequently right-sided (64.3%), compared to MGMT-unmethylated ones (43.1%) (*χ*^2^ = 4.1, *P* = 0.042), although the methylation percentage did not differ between tumours located in the two hemispheres. No statistically significant differences in the lobar distribution of GBMs, according to the MGMT methylation status and percentage, were found. The distribution frequency maps for both MGMT-unmethylated and MGMT-methylated GBMs are shown in Fig. 1.

### MGMT promoter methylation and GBM-network involvement

The repeated measures ANOVA showed that in both GMNs and WMNs models, GBMs were distributed unequally across different functional networks (Wilks’ lambda’s *F* = 29 with *P* < 0.001 and Wilks’ lambda’s *F* = 2002 with *P* < 0.001, respectively), as demonstrated in our previous study.^39^ However, the combined contribution of the MGMT methylation status covariate did not significantly explain differences in such distributions (Wilks’ lambda’s *F* = 1.6 with *P* = 0.07 and Wilks’ lambda’s *F* = 0.9 with *P* = 0.5, respectively).

### MGMT promoter methylation according to cortex, deep grey matter and white matter involvement

**Figure 2:**
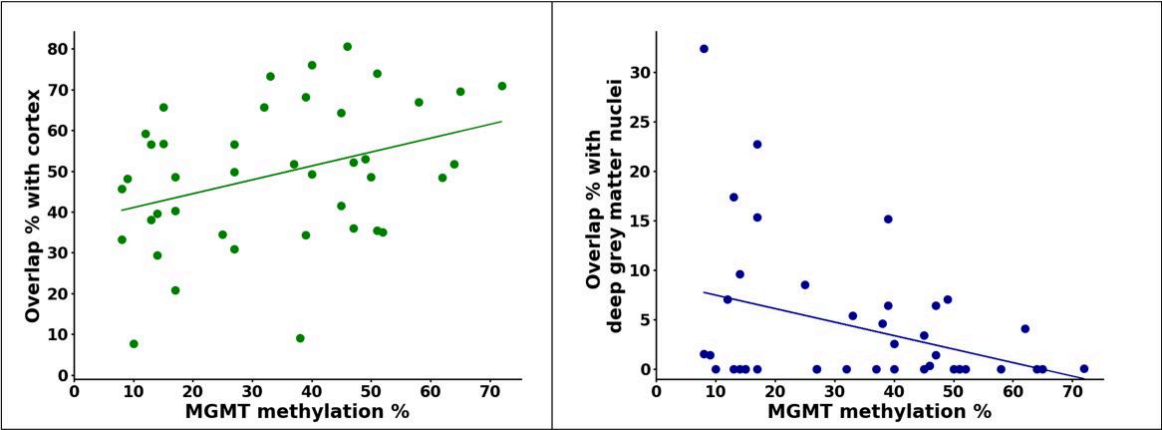
**Linear correlation between MGMT promoter methylation percentage and GBM core overlap percentage with cortex and deep grey matter nuclei**. The scatterplots show significant correlations with both cortex (Pearson correlation’s *R* = 0.36, *P* = 0.021, *N* = 41) and deep grey matter nuclei (Pearson correlation’s *R* = −0.35, *P* = 0.025, *N* = 41), respectively. Each dot represents a patient with an MGMT-methylated GBM.

While MGMT methylation status was not associated with cortical, WM or deep grey matter nuclei involvement, the MGMT methylation percentage, among MGMT-methylated patients, positively correlated with the percentage of GBM core overlapping the cortical grey matter (*R* = 0.36, *P* = 0.021; see Fig. 2) and negatively correlated with the involvement of deep grey matter nuclei (*R* = −0.35, *P* = 0.025; see Fig. 2), while it was not significantly associated with the WM involvement percentage (*R* = −0.21, *P* = 0.19). These results were also confirmed by partial correlations, correcting for tumour core volume (*R* = 0.35, *P* = 0.026, for cortical grey matter; *R* = −0.37, *P* = 0.017, for deep grey matter nuclei). Nonetheless, the absolute volume of overlap between GBM core and cortical/subcortical structures was not associated with the MGMT methylation status or percentage.

### Glioblastoma location and MGMT promoter methylation percentage: voxel-wise analysis

At the voxel-wise level, no significant topological differences between MGMT-unmethylated and MGMT-methylated GBMs, as well as between highly and lowly MGMT-methylated tumours, were found. Likewise, no involved anatomical location was linearly associated with the percentage of MGMT promoter methylation. Such results were unchanged after correction for GBM core volume, as well as after flipping all lesions to the same side.

## Discussion

In this study, we corroborated at the voxel-wise level the recent evidence that GBM topology does not depend on the categorical status of MGMT promoter methylation. Nonetheless, we also showed for the first time that the percentage of MGMT promoter methylation is higher when the GBM core is mainly located within the brain cortex and vice versa for the deep grey matter nuclei localization. Moreover, we excluded relationships between the status/percentage of MGMT promoter methylation and any other potential anatomical or topological distribution patterns, according to volume, side, lobar and functional network involvement. The only exception was a higher number of MGMT-methylated GBMs in the right hemisphere, partially conflicting with previous results^14-17^ and with the finding that the percentage of methylation was not different between the left and right hemispheres. In addition, recent studies with higher patient samples disproved those findings reported by the aforementioned studies, due to the lack of significant lateralization.^19-24^

Previous literature on this topic is somehow controversial, since, while two studies from the same research group showed a higher frequency of left temporal lobe involvement in MGMT-methylated GBMs,^14,15^ another one provided opposite results, without evidence of lateralization.^16^ When considering the quantitative level of MGMT methylation, a study reported that GBMs with low immunohistochemical MGMT expression were more frequent in the right parietal-temporal lobe, in contrast to highly MGMT-expressing tumours, whose occurrence prevailed in the left frontal lobe.^17^ On the other hand, other works did not show significant associations with anatomical location,^19,20^ in line with other recent studies based on voxel-based statistical techniques.^21-24^

Among the aforementioned studies, only four exclusively included IDH wild-type GBMs.^18,21-23^ While the first one focused on the relationship with the SVZ zone, the latter three were voxel-wise-based and, remarkably, in none of them associations with specific anatomical locations were found. It is thus possible that previously identified associations were driven by different molecular factors belonging to biologically distinct entities and that were however included in the same cohort prior to the recent conceptualizations of the 2016 and 2021 WHO classifications. In fact, IDH mutational status in low-grade gliomas is now known to be related to tumour location.^41^ Additionally, a more recent study employing the updated classification highlighted the presence of a gradient of glioma-related genes’ mRNA expression (among which MGMT was included) across the brain cortex, through principal component analysis. Nonetheless, the use of such dimensionality reduction technique might imply that the results derive from the contribution of multiple genes, rather than from MGMT expression alone.^42^

Regarding the relationship between the percentage of MGMT methylation and cortical/subcortical localization, our results were coherent with Han *et al*.’s finding that MGMT-methylated GBMs are associated with a more frequent sparing of the subventricular zone, although they did not investigate specific anatomical or lobar locations of tumours.^18^ Previous works have pointed out the lower survival of patients harbouring SVZ-contacting GBMs,^43-47^ which is coherent with our finding that deep-seated tumours have a significantly lower percentage of MGMT promoter methylation. Despite the surgical approach in ‘cortical’ GBM may usually reach higher levels of resection,^48^ previous results suggest that the role of MGMT methylation as a survival predictor remains independent of the extent of surgery.^49^ Delving into the possible mechanisms underlying the main result of the present study, previous works have shown distinct healthy brain DNA methylation patterns, in non-neuronal and neuronal cells, as well as between cortex and white matter.^50,51^ Moreover, recent literature suggests that the healthy brain regions most affected by glioma show higher expression of certain cancer-related genes,^31^ and advanced the existence of a ‘seed and soil’ hypothesis for glioma, in which tumours with a specific genetic signature tend to be located in regions with similar gene expression.^42^ On the basis of these findings and the known impact of tissue microenvironmental factors on brain tumour epigenetics,^52^ one could hypothesize that either the extent of MGMT methylation could be influenced by the epigenetics and genetics of the surrounding brain tissue or that gliomas with a specific methylation profile tend to preferentially arise in ‘permissive’ brain regions. Nonetheless, the precise biological mechanisms underlying this ‘gradient’ of cortico-subcortical MGMT methylation percentage are still unknown, but these observations should be considered in future studies addressing this issue.

It is worth mentioning that GBM is mainly a white matter disease, and when we refer to ‘cortical’ GBMs in the present study, we identify those GBMs (also encompassing the white matter) with a higher overlap with the cortex.^53^

We believe that the investigation of these latter aspects is indeed relevant, also in light of the recently published evidence suggesting increased survival rates with higher MGMT methylation percentages. More specifically, the authors found a statistically significant 2-year OS difference between patients with MGMT methylation below and above 15% (18.3 and 51.8%).^30^ In the same study, the median OS non-linearly increased with higher MGMT methylation percentage ranges: 14.8 months in the 0–4% range, 18.9 months in the 4–40% range and 29.9 months in the 40–100% range.

The present study is limited by the fact that the sample size is relatively low for the voxel-wise analysis; thus, we cannot exclude that larger cohorts may disclose some specific cortical associations.

## Data Availability

The original data generated throughout the course of this study, which is not accessible in the publication, will be made available personally upon reasonable request. In-house codes generated and used to perform some of the analyses of the present work are available in an online repository (https://github.com/GiulioSans/MGMT_analyses.git).
